# Impact of micronutrient fortification of yoghurt on micronutrient status markers and growth – a randomized double blind controlled trial among school children in Bangladesh

**DOI:** 10.1186/1471-2458-13-514

**Published:** 2013-05-28

**Authors:** Sunil Sazawal, AKM Ahsan Habib, Usha Dhingra, Arup Dutta, Pratibha Dhingra, Archana Sarkar, Saikat Deb, Jahangir Alam, Asmaul Husna, Robert E Black

**Affiliations:** 1 Department of International Health, Johns Hopkins Bloomberg School of Public Health, Baltimore, MD USA; 2 Center for Micronutrient Research, Department of Biochemistry, Annamalai University, Tamil Nadu, India; 3 Department of Community Medicine, Shaheed Ziaur Rahman Medical College, Bogra, Bangladesh

**Keywords:** Micronutrient fortification, Retinol binding protein, Iodine, Bangladesh, Zinc

## Abstract

**Background:**

Interventions providing foods fortified with multiple micronutrients can be a cost-effective and sustainable strategy to improve micronutrient status and physical growth of school children. We evaluated the effect of micronutrient-fortified yoghurt on the biochemical status of important micronutrients (iron, zinc, iodine, vitamin A) as well as growth indicators among school children in Bogra district of Bangladesh.

**Methods:**

In a double-masked randomized controlled trial (RCT) conducted in 4 primary schools, 1010 children from classes 1–4 (age 6–9 years) were randomly allocated to receive either micronutrient fortified yoghurt (FY, n = 501) or non-fortified yoghurt (NFY, n = 509). For one year, children were fed with 60 g yoghurt everyday providing 30% RDA for iron, zinc, iodine and vitamin A. Anthropometric measurements and blood/urine samples were collected at base-, mid- and end-line. All children (FY, n = 278, NFY, n = 293) consenting for the end-line blood sample were included in the present analyses.

**Results:**

Both groups were comparable at baseline for socio-economic status variables, micronutrient status markers and anthropometry measures. Compliance was similar in both the groups. At baseline 53.4% of the population was anemic; 2.1% was iron deficient (ferritin <15.0 μg/L and TfR > 8.3 mg/L). Children in the FY group showed improvement in Hb (mean difference: 1.5; 95% CI: 0.4-2.5; p = 0.006) as compared to NFY group. Retinol binding protein (mean diff: 0.05; 95% CI: 0.002-0.09; p = 0.04) and iodine levels (mean difference: 39.87; 95% CI: 20.39-59.35; p < 0.001) decreased between base and end-line but the decrease was significantly less in the FY group. Compared to NFY, the FY group had better height gain velocity (mean diff: 0.32; 95% CI: 0.05-0.60; p = 0.02) and height-for-age z-scores (mean diff: 0.18; 95% CI: 0.02-0.33; p = 0.03). There was no difference in weight gain velocity, weight-for-age z-scores or Body Mass Index z-scores.

**Conclusion:**

In the absence of iron deficiency at baseline the impact on iron status would not be expected to be observed and hence cannot be evaluated. Improved Hb concentrations in the absence of a change in iron status suggest improved utilization of iron possibly due to vitamin A and zinc availability. Fortification improved height gain without affecting weight gain.

**Trial registration:**

ClinicalTrial.gov: NCT00980733

## Background

Micronutrient malnutrition especially of iron, zinc, vitamin A and iodine is recognized as an important public health problem affecting more than 2 billion people worldwide [1]. Left untreated, even sub-clinical micronutrient malnutrition has significant negative consequences on health and economic development. As many as a third of the world’s population do not meet their physical and intellectual potential because of clinical and sub-clinical vitamin and mineral deficiencies [2]. Concern for micronutrient deficiency is particularly high among children as there is an increased nutritional demand among them due to growth spurts and high physical activity. The full genetic potential of the child for physical growth and mental development may be compromised due to sub clinical deficiencies of micronutrients, making them more vulnerable to develop frequent and more severe common day-to-day infections thus triggering a vicious cycle of under nutrition and recurrent infections [3-5]. Almost two-thirds of the deaths of children around the world are directly or indirectly associated with nutritional deficiencies [6].

Rural Bangladeshi populations, particularly children, are also at high risk of micronutrient deficiencies particularly vitamin A, iron, iodine and zinc, due to the consumption of a predominantly rice-based diet with few animal-source foods, iodine/zinc deficient soil [7], lack of access to safe drinking water, inadequate or nonexistent sanitation facilities and presence of infectious diseases and/or parasitic infection [8]. In Bangladesh, approximately 40 percent of children (6–15 years aged) have anemia with 6.9 percent of them having iron deficiency [9]. Thirty four percent of Bangladeshi children are iodine deficient with another 21.7 percent suffer from vitamin A deficiency [10]. A survey conducted in 2004–2005 showed that the prevalence of goiter among children 6 to 12 years old was 6.2% [11].

Supplementation based programs had limited success for iron deficiency, therefore in order to alleviate micronutrient deficiencies, WHO has recognized that future strategies should focus on identifying methods to deliver essential micronutrients such as iron, zinc, vitamin A, and iodine using suitable food vehicles [12]. There is increasing body of evidence suggesting micronutrient interventions might benefit the health and development of school age children and that multiple micronutrients might be more effective than single micronutrient. Fortifying foods with essential micronutrients is, thus a practical and cost effective strategy to improve nutrition status [13]. This has also been shown by a systematic review [5] for impact of fortification on improvement in micronutrient status and reduction in anemia among school children.

It is well recognized that given varied food preferences and consumption, multiple vehicles will need to be used in a practical community setup. Typically, locally produced and commonly consumed foods are the best candidates. In Bangladesh, yoghurt is a readily available and consumed food. Based on this premise, Grameen in Bangladesh in collaboration with social responsibility division of Dannon established non-profit social sector factory to produce yoghurt at low cost for local consumption in Bogra, Bangladesh. Global Alliance for Improved Nutrition (GAIN) used this opportunity to utilize locally produced yoghurt as a vehicle for delivery of 4 important micronutrients and thus the test intervention of this trial with 30% RDA of iron, zinc, vitamin A and iodine was developed.

In order to evaluate the efficacy of this intervention, we undertook a community-based, doubled-masked randomized trial in Bogra district, wherein children (aged 6–9 years) fed with micronutrient fortified yoghurt (30% RDA of iron, zinc, vitamin A and iodine) were compared to children fed with non-fortified yoghurt for one year on micronutrient status indicators (iron, zinc, vitamin A and iodine) and physical growth. This was based on the hypothesis that consumption of micronutrient fortified yoghurt by the school children over a period of one year would have a positive impact on their micronutrient status markers and physical growth indicators.

## Methods

### Study population and setting

The study was conducted between June 2008 and March 2010 in primary schools of Gabtali town of Bogra district in the Rajshahi Division, Northern Bangladesh. We selected schools in Gabtali town because of its close proximity to a yoghurt factory. Six primary schools from Bogra district were selected for the study. Of these 2 schools were assigned as pure control (no yoghurt was provided to the children and data from these two schools have not been used in the present analysis). The remaining four primary schools were randomly selected as yoghurt schools (fortified yoghurt and non fortified yoghurt). The study area had similar geographical characteristics to the rest of the plain lands in Bangladesh, including high population density, fertile agricultural land, low risk of malaria and minimal hookworm infection [14].

### Ethics

The human research and ethical review committees at the Johns Hopkins University, USA, Shaheed Zia-ur Rahman Medical College, Bangladesh and National Research Council of Bangladesh approved the study protocol.

### Sample size

Pretrial sample size estimation (based on assumptions from previous data), 400 children were planned to be enrolled and this sample size was estimated to, with an alpha of 0.05 and power of 90%, provide ability to detect a change in height by 2.56 cm and weight by 930 g over the baseline weight and height measurements; detect a change in mean hemoglobin (Hb) level by 2.6 g/L over the baseline Hb; detect reduction in iron deficiency anemia by 38% and zinc deficiency by 37%. The sample size was provided for 5% attrition. However, due to practical reasons we had to enroll every child in class in yoghurt groups, for which we amended the protocol and thereby enrolled 500 children per group. This worked well as the attrition due to refusal for blood sampling was also higher than anticipated 5%.

### Consent and enrollment

Before the start of the study, the purpose and details of the study was explained in a meeting to all concerned parents and school officials. The possible risks and benefits associated were read out and explained to the parents in their local language, and informed written consent sought from the parents of each of the selected children prior to the enrollment in the study. For practical reasons, all the children of the class 1–4 were invited to take part in the study. The inclusion criteria for enrollment into the study was children aged 6 to 9 years attending selected schools, who were likely to remain in the same school and parents providing consent. Children with severe malnutrition needing nutritional rehabilitation or chronic/severe illness requiring hospitalization or special treatment were excluded and referred for treatment. After obtaining consent from parents and assent from children, children were enrolled and scheduled to visit clinics for baseline assessments.

### Masking and randomization

Children were randomized by their enrollment serial number into either the micronutrient fortified yoghurt (FY) group or non-fortified yoghurt (NFY) group that would receive identical yoghurt with no added micronutrients. Using in-house computer software, a random sequence of group codes with a permuted block length of 6 was generated to randomly allocate the individual child to one of the two yoghurt groups (NFY group or FY group). Group codes from 1 to 6 were used to identify the fortified and non-fortified yoghurt. The codes of the groups were not known to the investigators, field team, teachers, children or anyone involved in the study during the field implementation. Cups were prepared and labeled with group codes a day in advance at a factory in Bogra. The yoghurt for the two intervention groups was identical in packaging, appearance, taste and smell. Children in both the groups were followed up for 12 months.

### Yoghurt distribution and follow-up

Every day in the morning, the yoghurt cups were collected from the factory and the delivery containers were insulated with ice packs to maintain the temperature and quality of the yoghurt during transportation and delivery. The yoghurt delivery team distributed the yoghurt in the class by code allocated to the child during randomization. The assistant verified the child’s identity (e.g. child’s name, name of father and mother). Children allocated to the yoghurt groups received one cup of the yoghurt (60 g) daily during the lunch break of the school for one year. The feeding session was strictly monitored and supervised by the field workers and class teachers and the compliance to the intervention was recorded in compliance record forms. A separate list was prepared for children who were absent from school and their respective yoghurt cups were delivered at home in the afternoon. In case of absenteeism due to sickness, morbidity information of the child was recorded in a data record sheet. During holidays, yoghurt was distributed at pre-scheduled points near the child’s home.

Children were informed beforehand about their respective delivery points. For children who did not turn up at these locations, yoghurt was taken to their homes. The detailed composition of fortified yoghurt is presented in Table 1.

**Table 1:** Composition of fortified yogurt provided to the study participants*

**Nutritional composition**	**Per 60 g**	**% RDA**
Energy (Kcal)	71.6	-
Protein (g)	2.3	-
Lipids (g)	2.5	-
Carbohydrates (g)	10.1	-
Of which sugars (g)	3.8	-
Calcium (mg)	85	18.4%
Phosphorus (mg)	67	18.7%
Iron (mg)	3.3	30.0%
Zinc (mg)	3.0	30.0%
Iodine (μg)	40	30.0%
Vitamin A (μg)	140	30.0%

*In addition contains: Whole milk, Date molasse, Sugar starch, Aspartame, Acesulfam K, Danone Yogurt culture, micronutrient premix.

### Data collection

#### Socio-demographic assessments

Data concerning socio-economic status (SES) was collected at baseline only. During the survey, parents or caregivers were interviewed about socio-demographic characteristics of the children (age, sex, illness history and medical supplements) and mothers (age, education, family size, and household SES) using standardized questionnaires by trained field workers.

#### Blood/urine sample collection and anthropometric measurements

Anthropometric data and blood/urine samples were collected at base- mid- and the end-line from all children belonging to FY and NFY groups. In the selected schools at base-, mid- and end-line, clinics were established to carry out a medical examination, collect blood/ urine samples and measure the height and weight of children.

A unique bar-coded ID for each child was used and a clinic kit specific for that child was issued after verifying the child’s identity. A non-fasting venous blood sample (~5 mL) was drawn by a trained technician using trace element-free syringes. From this sample, 2 mL of blood was immediately transferred into an ethylenediaminetetraacetic acid (EDTA) vial to obtain a detailed hemogram and the rest was centrifuged at 4000 rpm for 20 minutes. Plasma was separated within 15 minutes of blood collection, and aliquots were transferred into 2 trace element-free micro-centrifuge tubes and stored at −20°C for further micronutrient analysis. For all blood sample collection procedures, trace element-free tubes and pipettes were used and samples were protected against sunlight. Casual urine samples were collected in 10 mL capped polypropylene tubes and stored at −20°C for later urinary iodine analysis. Plasma samples were analyzed for markers of iron (transferrin receptors (TfR) and ferritin), retinol binding protein (RBP), zinc status and acute phase proteins: C-reactive protein (CRP) and α-1 acid glycoprotein (AGP).

Weight was measured to the nearest 10 g using an electronic scale (Kantewala, Delhi, India) by two independent observers while the children were wearing their school uniforms. The scale was checked for accuracy with standard weights after about every 25 measures. Height was measured to the nearest 0.1 cm without shoes using non-stretchable microtoise tape posted flat against a wall.

#### Laboratory analysis of blood and urine samples

A small aliquot of blood was analyzed on an automated hematology analyzer (Sysmex XT-1800i analyzer, Japan) with WBC 5-part differential at the Popular Diagnostic Center Limited, Bogra, Bangladesh on the same day as the blood was collected. Routine hematology controls were used for quality control (Sysmex hematology controls, Japan). Double sandwich ELISA was used to analyze plasma ferritin, TfR, vitamin A (RBP), CRP and AGP markers at DBS-Tech, Willstaett, Germany [15]. Plasma zinc estimations were performed by standard methods using an atomic absorption spectrometer (AAS 800-Perkin Elmer) at the laboratory facility of the Center for Micronutrient Research, Annamalai, India [16]. The urinary samples were processed for estimation of urinary iodine using the modified Sandell-Kolthoff microplate method [17] at the WHO reference laboratory at the All India Institute of Medical Sciences (AIIMS), New Delhi. Assay precision was evaluated on repeated analysis of pooled plasma and urine samples. Ten percent of samples of each parameter were measured in duplicate. The within-assay variability of plasma ferritin, TfR, RBP, CRP, AGP, zinc, and urinary iodine was <6% and between-assay variability was < 10%.

#### Definitions

Anemia was defined as Hb concentration < =115.0 g/L for children aged 6–9 years old [18]. Plasma ferritin concentrations <15 μg/L was defined as depleted iron stores [18]. Iron deficiency anemia (IDA) was defined as Hb < 115.0 g/L and plasma ferritin <15 μg/L [18]. Plasma TfR concentration >8.3 mg/L was considered an indication of tissue iron deficiency [19]. Body iron was calculated using the equation: body iron (mg/kg) = − [log(TfR/ferritin ratio) -2.8229]/0.1207 [20].

Vitamin A deficiency was defined as plasma RBP concentrations <0.70 μmol/L [21]. Zinc deficiency was defined as plasma zinc concentration <9.945 μmol**/**L for blood samples taken from non-fasting subjects [16,22]. Low urinary iodine excretion (UIE) was defined as urinary iodine concentration <100 μg/L [18]. A plasma concentration of CRP (>5 mg/L) and/ or AGP (>1g/L) was considered an indication of an acute phase response [15].

#### Statistical analyses

Visual basic and Oracle 8i was used for data entry and management. Data were entered daily and checked consistently for errors. The analyses were performed in STATA 10.0 (Stata Corp., College Station, Texas, USA). Anthropometric Z-scores were calculated using WHO standards [23]. Intent-to-treat analysis was performed and all children (FY-group 278, NFY-group 293) consenting for the end-study blood sample were included in analyses. For the primary analysis the authors used alphabetical codes for the groups as they were still blinded to the real group identity. The groups were assessed for comparability at baseline. The statistical significance for the group differences of continuous variables was established using a Student’s t-test, and of categorical variables by chi-square test. Significance was considered as *P*-values ≤0.05. Paired t-test was used to determine if there were significant mean differences in the markers for micronutrient status and growth parameters within each group before and after intervention and 95% CI were calculated for these differences. Further, paired analysis was performed to compare the change in status of micronutrient markers at baseline and end study between two groups. For the growth analysis corrected for baseline, regression models were fitted with the end study measurements as the dependent variable, group allocation and baseline measurement as the independent variable.

## Results

### Participants

**Figure 1:**
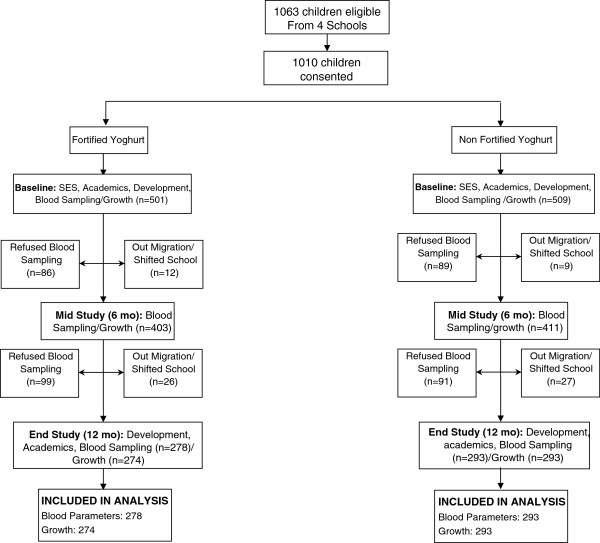
Trial profile.

Figure 1 shows the trial profile. Of the 1063 eligible children from 4 primary schools, 1010 provided consent. The enrolled children were randomly allocated to receive either micronutrient-fortified yoghurt (FY group; n = 501) or the same yoghurt without added micronutrients (NFY group; n = 509). The dropout rate (18% at mid study and 22% at end study) was similar between the study groups, and the main reasons for dropout were refused blood sampling, relocation out of the study area, change of school and a very small proportion of children who did not like yoghurt (Figure 1). Baseline data available for the subjects who were lost to follow up did not differ from data for those included in the present analysis (data not shown).

### Baseline data

The baseline profile of the study participants by intervention group is shown in Tables 2 and 3. Both the fortified and non-fortified groups were comparable for socio-economic variables (Table 2), micronutrient status and anthropometry (Table 3) at baseline. The mean age of the participants was 7.0 ± 0.5 years and 52.4% were girls. The mean Hb level was 114.7 g/L while mean red cell distribution width was 14.4% (Table 3). The total prevalence of anemia was 53.4% (Hb < =115.0 g/L) and was not significantly different between boys and girls (Chi-square test, P > 0.05). At baseline 2.1% children were iron deficient (ferritin <15.0 μg/L and TfR > 8.3 mg/L) and this was not affected, even if the children with infection were removed from the analysis. The mean concentration of serum retinol binding protein (RBP) was 1.24 ± 0.29 μmol/L. Children with RBP concentration <0.7 μmol/L (vitamin A deficiency) and between 0.7-1.05 μmol/L (marginal vitamin A deficiency) was 1.1% and 23.6%, respectively. The mean urinary iodine level was 171.6 μg/dL and the proportion of children <100 μg/dL was 27.6%. The zinc status was less than optimal; the mean plasma zinc concentration was 9.17 μmol/L and 70% children had zinc levels <9.945 μmol**/**L. The concentrations of plasma ferritin and RBP of those school children with elevated acute phase proteins (CRP > 5 mg/L and/or AGP >1 g/L) compared to those with normal concentrations at the time of the baseline blood sampling were different. Those in the fortified yoghurt and non-fortified yoghurt group had significantly higher ferritin concentrations (p = 0.006 and 0.02 respectively) and significantly lower RBP concentrations (p = 0.13 and p = 0.00, respectively) when the acute phase proteins were elevated (Additional file 1: Table S5). Other blood parameters were not affected by the presence of elevated acute phase proteins. However the results in the subgroup with no inflammation (89.1% of the sample) were not different from overall results. The prevalence of stunting, underweight and BMIZ scores - < 2 Z were 22.2%, 42.2% and 31.4% respectively. None of these prevalence were significantly different between boys and girls (Chi-square test, P > 0.05).

**Table 2:** Baseline comparison for SES and other variables among two groups

	**Fortified yoghurt**	**Non fortified yoghurt**
	**(n = 501)**	**(n = 509)**
Gender		
Boys	42.2	43.1
Type of house		
Kacha	72.0	76.4
Pucca	10.1	9.5
Kacha-Pucca	17.9	14.1
Own House	96.2	92.5
Family Type		
Nuclear	92.8	94.0
Mother age	31.6 ± 6.4	31.3 ± 5.9
(mean ± SD)		
Mother Education		
Illiterate	24.6	23.6
Primary	42.2	42.8
Secondary	24.6	22.7
College	8.7	10.9
Father Education		
Illiterate	26.6	26.1
Primary	35.5	39.4
Secondary	18.8	15.5
College	18.5	17.8
Mother employment		
Homemaker	94.2	93.7
Father employment		
Business	32.9	37.6
Farming	28.0	24.7
Father income (inRupees)		
No Income	0.3	0.9
1000-2000	26.9	27.3
3000-5000	55.2	53.2
6000-10000	10.1	10.1
>10000	6.9	7.5

All values are in percentages unless specified.

**Table 3:** Baseline comparisons for blood and growth parameters between fortified and non-fortified yoghurt groups

**Variables**	**Fortified yoghurt (n = 278)**	**Non-fortified yoghurt (n = 293)**
	**Mean ± SD**	**Mean ± SD**
***Growth parameters *weight (kg)**	21.94 ± 4.72	21.39 ± 4.52
Height (cm)	124.0 ± 9.09	122.8 ± 8.90
WAZ	−1.68 ± 1.32	−1.82 ± 1.42
< −2.0 (n, %)	114 (41.6)	125 (42.7)
HAZ	−1.01 ± 1.20	−1.17 ± 1.17
<−2.0 (n, %)	57 (20.8)	69 (23.5)
BMIZ	−1.59 ± 1.25	−1.80 ± 3.28
<−2.0 (n, %)	92 (33.6)	86 (29.4)
***Iron status markers *hemoglobin(g/L)**	114.7 ± 8.6	114.7 ± 8.8
Red cell width (%)	14.39 ± 2.77	14.26 ± 2.27
Serum Ferritin (μg/L)	68.28 ± 35.99	70.03 ± 39.25
Serum Transferrin receptor (mg/L)	5.69 ± 1.17	5.79 ± 1.21
Body Iron Store (mg/Kg Bodywt)	7.03 ± 2.23	6.99 ± 2.25
Retinol binding Protein (μmol/L)	1.25 ± 0.30	1.22 ± 0.27
Zinc (μmol/L)	9.11 ± 3.30	9.22 ± 3.65
Iodine (μg/L)	160.71 ± 93.12	182.42 ± 102.96
***Infection defined as***		
CRP(>5 mg/L) (incubation) (n, %)	8 (2.7)	6 (2.0)
AGP (>1 g/dL) (Convalescence) (n, %)	26 (9.4)	33 (11.3)
AGP (>1 g/dL) and/or CRP (>5 mg/L)	29 (10.4)	33 (11.3)

### Compliance

As the feeding sessions were monitored, the compliance (adherence to intervention) was assessed by collecting information on consumption and recording the remaining quantity of yoghurt (not consumed by the children) during the one-year intervention period. The compliance of the children for intervention in both the groups was ~97.0 percent and was similar in both groups.

### Effect on micronutrient status and micronutrient deficiency prevalence

Consumption of fortified yoghurt did not affect the iron status indicators and body iron stores. However, children in the FY-group showed improvement in Hb levels (0.8 g/L; mean difference: 1.5; 95% CI: 0.4-2.5; p = 0.006) compared to NFY-group (Table 4). Although concentrations of both RBP and iodine went down from baseline, compared to the NFY group, the RBP and iodine concentrations were significantly better in the children receiving fortified yoghurt, with estimated effect sizes of 0.05 μmol/L (p = 0.04) and 39.87 μg/dL (p = <0.001) respectively (Table 4). Severe-iodine deficiency was present in only 5.1 percent of children. Zinc status improved in the fortified group but the difference in mean zinc levels between the two groups was not statistically significant. Although mean zinc concentrations improved, they were still below the accepted cutoff for the age group at end-line (9.94 μmol/L). Furthermore, consumption of micronutrient fortified yoghurt reduced the worsening of vitamin A and iodine status that was observed in control group there by showing a significant difference in RBP levels and urinary iodine excretion. The concentrations of plasma ferritin and RBP of those school children with elevated acute phase proteins (CRP > 5 mg/L and/or AGP >1 g/L) compared to those with normal concentrations at the time of the end-line blood sampling were different (Fortified Yoghurt p = 0.00; p = 0.001 Non Fortified Yoghurt p = 0.04; p = 0.001) (Additional file 1: Table S6). Other blood parameters were not affected by the presence of elevated acute phase proteins. However the results in the subgroup with no inflammation (89.3% of the sample) were not different from overall results suggesting that inflammation did not contribute to observed results for ferritin and RBP.

**Table 4:** Paired comparison for blood parameters between baseline and end study comparing fortified and non-fortified yoghurt groups

**Variable**	**Fortified yoghurt (n = 278) Mean ± SD**	**Non-fortified yoghurt (n = 293) Mean ± SD**	Mean Diff*	**95% CI**		**P value**
				**Lower**	**Upper**	
**Hb **(g/L)	0.8 ± 0.61	−0.6 ± 0.66	1.5	0.4	2.5	**0.006**
**Ferritin** (μg/L)	−0.49 ± 31.75	1.56 ± 27.35	−2.05	−6.91	2.82	0.41
**sTfR **(mg/L)	−0.008 ± 0.74	−0.02 ± 0.75	0.01	−0.11	0.14	0.86
**Body Iron Store**	−0.01 ± 1.53	0.08 ± 1.42	−0.09	−0.33	0.15	0.46
(mg/Kg B wt)						
**RBP **(μmol/L)	−0.01 ± 0.29	−0.06 ± 0.25	0.05	0.002	0.09	**0.04**
**Zinc **(μmol/L)	1.44 ± 4.37	1.24 ± 4.01	0.20	−0.57	0.98	0.61
**Iodine** (μg/L)	−42.6 ± 130.0	−82.5 ± 121.9	39.87	20.39	59.35	**0.001**

*Mean difference was calculated using paired analysis.

### Effect on physical growth

Compared to NFY-group, children in FY-group had better height gain velocity (5.46 cm/yr, mean difference: 0.32; 95% CI: 0.05-0.60;p = 0.02) and height-for-age z-scores (HAZ) (mean difference: 0.18; 95% CI: 0.02-0.33;p = 0.03). There was no difference in weight gain velocity, WAZ or BMIZ scores (Table 5). After correcting for baseline measurements, children in the FY group still had significantly better HAZ and height gain velocity than those in the NFY (Table 6).

**Table 5:** End study comparisons for growth parameters between fortified and non-fortified yoghurt groups

	**Fortified yoghurt (n = 274) Mean ± SD**	**Non-fortified yoghurt (n = 293) Mean ± SD**	Mean Diff*/Odds ratio	**95% CI**		**P value**
				**Lower**	**Upper**	
Weight velocity (kg/yr)	3.04 ± 1.73	3.06 ± 1.41	−0.02	−0.29	0.23	0.84
Height velocity (cm/yr)	5.46 ± 2.00	5.14 ± 1.24	0.32	0.05	0.60	0.02
WAZ	−1.51 ± 1.20	−1.54 ± 1.21	0.03	−0.14	0.20	0.68
<−2 (n, %)	93 (33.9)	96 (32.8)	0.77	1.05	0.74	1.50
HAZ	−1.00 ± 1.12	−1.17 ± 1.15	0.18	0.02	0.33	0.03
<−2 (n, %)	49 (17.9)	68 (23.2)	0.72	0.48	1.09	0.12
BMIZ	−1.28 ± 1.24	−1.17 ± 1.08	−0.10	−0.29	0.09	0.29
<−2 (n, %)	66 (24.1)	61 (20.8)	1.21	0.81	1.79	0.35

*Mean difference was calculated using paired analysis.

**Table 6:** Comparison for growth parameters between fortified and non-fortified yoghurt groups at end-study adjusted for baseline measurements

	**Fortified yoghurt (n = 274) Mean ± SD**	**Non-fortified yoghurt (n = 293) Mean ± SD**	Mean Diff*/Odds ratio	**95% CI**		**P value**
				**Lower**	**Upper**	
Weight velocity (kg/yr)	3.04 ± 1.73	3.06 ± 1.41	−0.02	−0.29	−0.23	0.84
Height velocity (cm/yr)	5.46 ± 2.00	5.14 ± 1.24	0.32	0.05	0.60	0.02
WAZ	0.22 ± 0.70	0.29 ± 0.77	−0.04	−0.14	0.07	0.50
<−2 (n, %)	93 (33.9)	96 (32.8)	1.22	0.72	2.06	0.47
HAZ	0.05 ± 0.40	0.008 ± 0.24	0.06	0.005	0.11	0.03
<−2 (n, %)	49 (17.9)	68 (23.2)	0.51	0.23	1.12	0.09
BMIZ	0.32 ± 0.88	0.63 ± 3.05	−0.14	−0.32	0.03	0.11
<−2 (n, %)	66 (24.1)	61 (20.8)	1.21	0.81	1.79	0.35

*Mean difference was calculated using paired analysis.

## Discussion

The study was designed to investigate whether regular consumption of micronutrient fortified yoghurt (delivering 30% RDA of zinc, iron, iodine and vitamin A) over a period of 1 year could improve the micronutrient status and physical growth of school children. To our knowledge, this is one of the first reports of a study that used yoghurt as a vehicle for delivery of micronutrients and evaluated the impact among school children in a double-blind RCT. Although consumption of fortified yoghurt did not result in improvement in iron status indicators, it did show a significant improvement in Hb, and an impact of iodine status indicated by significantly lower decline urinary iodine levels when compared to the NFY group. One year of fortification resulted in an improvement in linear growth with a statistically significant change in height velocity and HAZ. A systematic review [5], evaluated the effect of the multi-micronutrient (MMN) fortification of foods compared to unfortified foods on the micronutrient status of school children and measured a statistically significant improvement in micronutrient status in the intervention group compared to the control group, taking baseline values into account. Some studies have reported positive effects on growth but the overall effects on these outcomes are equivocal [5]. The response to fortification in the present study showed an improvement in Hb concentrations, even though no iron deficiency was present at baseline, and although RBP and iodine concentrations fell in both groups, they were significantly higher in the FY groups. A decline in the intervention group may be suggesting 30% RDA of selected micronutrients in the fortified yoghurt may not have been adequate to reverse decline in vitamin A and iodine concentrations in this population of school children.

In the present study, iron deficiency was not observed i.e. ferritin, TfR and estimated body iron stores were in normal range. Consistent with our finding, Winichagoon et al. [13] did not find an effect on iron status indicators after adding 5 g of elemental iron via multi-micronutrient (MMN) powder to the school lunches of Thai children. It has been demonstrated that the maximal inhibitory effect of calcium on iron absorption is reached at a level of ~300 mg of calcium [24]. The possibility of the calcium content having been responsible for lack of effect on iron status markers is very unlikely as the calcium content of yoghurt was less than 100 mg. However cannot be totally ruled out given the study design and fairly adequate iron status of the population and therefore a possibility of reduced absorption and potential for minor impact.

In this study, the prevalence of elevated plasma CRP and AGP was low; indicating the presence of ongoing or recent acute inflammation in the study population was low, however that may not rule out some chronic infections but then their presence would have caused anemia rather than contrary. Prevalence of hemoglobinopathy, another possible cause for high ferritin concentrations in the presence of anemia, needs consideration. Anemia was present in a little more than half of the children in both the groups at baseline with a mean Hb concentration of 114.7 g/L. But levels of Hb in this population and distribution of Hb do not favor hemoglobinopathy as a cause of high ferritin. In fact in population with much higher levels of anemia in Delhi contribution of hemoglobinopathy to levels of ferritin has not been demonstrated (Unpublished-data). There was a significant difference in Hb levels of 1.5 g/L between FY and NFY groups after one year of fortification. A similar effect on Hb status was recently reported among Kenyan subjects with HIV infection in whom MMN supplementation resulted in increased Hb concentrations only among those without signs of inflammation [25]. Although there are studies which found significant effects of multiple micronutrient fortification on concentrations of Hb, TfR, ferritin and on body iron stores [26], in the present study, the difference in Hb between the FY and NFY groups, despite the lack of difference in the iron status markers suggest better utilization of iron, which may have been caused by bio-available zinc and vitamin A. Vitamin A supplementation has been shown to increase erythropoietin (EPO) [27]. Retinoic acid, a vitamin A metabolite, has been reported to regulate the EPO gene, which, in turn enhances EPO production *in vitro* in an animal model [28]. Providing vitamin A in the fortified yoghurt may have increased the mobilization of iron from storage [29,30], resulting in an improvement in Hb concentration, although the mechanism of this phenomenon is still to be verified.

At baseline, some of the children were mild to moderately deficient in iodine. The population where the study was conducted were found to consume plain salt more than the iodized salt, although, the national salt iodization program of 1989 in Bangladesh is ongoing on. In our study, iodine and RBP decreased in both the groups between base- and end-line, but the decrease was significantly less in FY group, suggesting compliance to and success of intervention in delivering nutrients. Similarly, all five studies included in the systematic review also found a beneficial effect of MMN fortification on urinary iodine excretion levels [5].

Zinc status was less than the optimum at the baseline. Zinc status improved but the differences in mean zinc levels were not statistically significant and concentrations were still below the accepted cutoff for the age group at end-line (9.94 μmol/L). A review of six studies investigated the effect of MMNs (including zinc) in fortified food compared to unfortified food on zinc status, only two studies found a significant beneficial effect [13]. While other studies, did not find any effect on biochemical zinc status or deficiency [30-32]. Although dairy products have shown to increase zinc absorption [33], the sub-optimum effect of the zinc in the fortified yoghurt on zinc status could be due to the level of zinc added being too low, to a possible negative interaction between iron and zinc [34] or because plasma zinc may not be a good marker of zinc status. Indeed, recent studies have shown that iron inhibits zinc absorption [35] and vice versa. However, iron has little effect on zinc absorption when zinc-iron ratios are 1:1, which is similar to the ratio in our study [36]. Therefore, the interaction of iron and zinc from the fortified yoghurt should be further investigated and the dosage of zinc should be re-evaluated. Further, the beneficial effects on observed height gain, which are commonly attributable to zinc intake rather than iron [37,38] would argue for an improvement in zinc status which plasma zinc was not able to reflect.

One year of fortification resulted in an improvement in linear growth with a statistically significant change in height velocity and height for age Z- scores, but not on weight and BMIZ which was likely attributed to the synergetic effects of the micronutrients including the presence of zinc [39]. A meta-analysis on the effects of micronutrients on growth in children up to 18 years of age found that interventions with iron or vitamin A alone did not have an effect on either height or weight gain, whereas MMN interventions, 80% of which included vitamin A, iron, and zinc, significantly improved linear growth and also had a small (not statistically significant) positive effect on weight gain [40]. These findings would suggest that providing specific micronutrients results in a better height gain, and reversal of growth faltering over and above of macronutrients [26]. A systematic review that tested the impact of multiple micronutrients provided via fortification on the micronutrient status, growth, health, and cognitive development of school children showed mixed results i.e. no effect to a positive effect on growth. Two of seven studies found a significant beneficial effect on height gain, with mean differences of about 0.6 to 1.0 cm in height increments between the groups after 6 or 14 months of intervention [5]. Finally, improvement in Hb levels, and decline in the excretion of urinary iodine levels and improvement in growth points towards a global improvement in child’s health - a functional endpoint of multiple metabolic processes. This global improvement could be due to a combination of effects of individual constituents of the intervention and/or synergistic effects among the components.

### Limitation of the study

The results of the study need to be interpreted with consideration to the initial micronutrient status of the population. The children in this study did not have severe anemia or iron deficiency at baseline. Given the design of the study we cannot attribute effects to a specific component but only describe the collective impact of the specific micronutrients tested as the experimental intervention in this study.

## Conclusions

At baseline although 50% children had mild anemia, but there was no iron deficiency in the children. The children had normal mean RBP and iodine concentrations; however mean zinc concentrations were just below the accepted cutoff for the age group. Following 12 months of supplementation fortification, there was no improvement in iron status indicators; however, a significant improvement in hemoglobin levels was observed. Although plasma concentrations of iodine and RBP in both yoghurt groups fell over the 12 month period, concentrations of these in the FY decreased significantly less. One year of fortification resulted in an improvement in linear growth with a statistically significant change in height gain among the children of the FY group.

Future studies need to investigate a number of operational issues including the feasibility of distributing the product through different channels such as schools (as used in this study), and also through the private sector, using a social marketing approach.

## Abbreviations

RBP: Retinol binding protein; CRP: C-reactive protein; IDA: Iron deficiency anemia; MMN: Multi micronutrient; TfR: Transferrin receptor; NFY: Non fortified yoghurt; FY: fortified yoghurt; SES: Socio economic Status; Hb: Hemoglobin; WAZ: Weight for age z-scores; BMIZ: Body mass index z-scores; HAZ: Height for age Z-scores.

## Competing interests

GAIN- Global Alliance for Improved Nutrition funded the study and Grameen Danone Foods Ltd. Bogra, Bangladesh supplied the yoghurt. The company however did not have any role in study funding, design, data collection, analysis, decision to publish or preparation of the manuscript. The authors declare that they have no competing interests.

## Authors’ contribution

The authors’ responsibilities were as follows – SS, AKMAH, JA and REB: conceived and designed the study, directed the data analysis, and critically revised the manuscript for important intellectual content.; UD and AD: designed and developed data collection tools, supervised the data collection, analyzed data, interpreted the results and edited the paper; PD, AS, AH supervised the data collection and Training of staff and quality control. PD and SD prepared the draft paper and incorporated review changes in consultation with SS and UD. None of the authors was affiliated in any way with an entity involved with the manufacture or marketing of yoghurt. All authors read and approved the final manuscript.

## Pre-publication history

The pre-publication history for this paper can be accessed here:

http://www.biomedcentral.com/1471-2458/13/514/prepub

## Supplementary Material

Additional file 1: Table S1 Sample size estimations. **Table S2.** Using different cutoffs, the blood parameters (n, %) were compared between the fortified and non fortified yoghurt groups at base-line and end-line. **Table S3.** Baseline comparison for SES and other variables among two groups for the subset contributing to study. **Table S4.** Comparison for blood parameters between fortified and non-fortified yoghurt groups at end-line. **Table S5.** Concentrations of biomarkers in those with normal and elevated acute phase proteins at baseline. **Table S6.** Concentrations of biomarkers in those with normal and elevated acute phase proteins at end-line. Click here for file
