# Supplementary material for: Impact of micronutrient fortification of yoghurt on micronutrient status markers and growth – a randomized double blind controlled trial among school children in Bangladesh
Source: BMC Public Health. 2013 May 28;13:514. doi: 10.1186/1471-2458-13-514 (PMC3671231; doi:10.1186/1471-2458-13-514)
Supplement: Additional file 1: Table S1 — Sample size estimations. Table S2. Using different cutoffs, the blood parameters (n, %) were compared between the fortified and non fortified yoghurt groups at base-line and end-line. Table S3. Baseline comparison for SES and other variables among two groups for the subset contributing to study. Table S4. Comparison for blood parameters between fortified and non-fortified yoghurt groups at end-line. Table S5. Concentrations of biomarkers in those with normal and elevated acute phase proteins at baseline. Table S6. Concentrations of biomarkers in those with normal and elevated acute phase proteins at end-line. [file 1471-2458-13-514-S1.docx]

**Supplementary Table 1: Sample size estimations**

| **Outcome** | **Control Group**  **assumptions** | **Performance**  **Targeted** | **Number of children per group with power of**  (with 5% attrition rate) | |
| --- | --- | --- | --- | --- |
|  |  |  | **90%** | **80%** |
| **Status Indicators** |  |  |  |  |
| Proportion Iron deficient anemia | 31% | Reduction  38% (90% power)  33.5% (80% power) | 380 | 375 |
| Difference in Hb g/L | Mean116 SD(11) | Mean increase in  2.6 (90% power)  2.2 (80% power) | 380 | 380 |
| Proportion Zinc Deficient | 33% | Reduction  37% (90% power)  32.5% (80% power) | 379 | 377 |
| **Growth**  (Am J Clin Nutr 2003;78:406-13) |  |  |  |  |
| Height (cm) | Mean 114.9,SD(10.8) | Mean increase in  2.56 cm (90% power)  2.2 cm (80% power) | 374 | 378 |
| Weight (Kg) | Mean 18.3 SD(3.95) | Mean increase in  930 g (90% Power)  810 g (80% Power) | 379 | 373 |

**Supplementary Table 2: Using different cutoffs, the blood parameters (n, %) were compared between the fortified and non fortified yoghurt groups at base-line and end-line**

| **Variable** | **Fortified yoghurt**  **n (%)** | **Non fortified yoghurt**  **n (%)** | **OR 95% CI** | **P value** |
| --- | --- | --- | --- | --- |
| **Anemia**  **(Hb ≤ 115 g/L)** |  |  |  |  |
| Baseline | 147 (52.9) | 158 (53.9) | 0.96 (0.69-1.33) | 0.80 |
| End study | 141 (50.7) | 153 (52.2) | 0.94 (0.68-1.31) | 0.72 |
| **Ferritin < 15 µg/L** |  |  |  |  |
| Baseline | 3 (1.1) | 6 (2.1) | 0.52 (0.13-2.11) | 0.35 |
| End study | 1 (0.4) | 6 (2.0) | 0.17 (0.02-1.44) | 0.07 |
| **sTfR> 8.3 mg/L** |  |  |  |  |
| Baseline | 6 (2.2) | 9 (3.1) | 0.69 (0.24-1.96) | 0.48 |
| End study | 4 (1.4) | 8 (2.8) | 0.52 (0.15-1.73) | 0.27 |
| **Zinc <9.94 µmol/L** |  |  |  |  |
| Baseline | 188 (67.9) | 213 (72.7) | 0.79 (0.55-1.14) | 0.21 |
| End study | 134 (48.4) | 144 (49.3) | 0.96 (0.69-1.34) | 0.82 |
| **RBP <0.70 µmol/L** |  |  |  |  |
| Baseline | 1 (0.4) | 5 (1.7) | 0.21 (0.00-1.35) | 0.11 |
| End study | 1 (0.4) | 9 (3.1) | 0.11 (0.00-0.70) | 0.01 |
| **Iodine < 100.0** µg/L |  |  |  |  |
| Baseline | 76 (28.5) | 73 (26.1) | 1.13 (0.77-1.64) | 0.53 |
| End study | 142 (56.1) | 166 (62.6) | 0.76 (0.54-1.08) | 0.13 |
| **Incubation (elevated CRP only)**  **(CRP>5 mg/l)** |  |  |  |  |
| Baseline | 8 (2.9) | 6 (2.1) | 1.42 (0.49-4.14) | 0.52 |
| End study | 5 (1.8) | 10 (3.4) | 0.52 (0.18-1.54) | 0.22 |
| **Convalescence (elevated AGP only)**  **(AGP >1 g/L)** |  |  |  |  |
| Baseline | 26 (9.4) | 33 (11.3) | 0.81 (0.47-1.40) | 0.45 |
| End study | 31 (11.2) | 25 (8.5) | 1.37 (0.79-2.39) | 0.26 |
| **Infection – (elevated CRP or AGP)**  **CRP>5 mg/l and/or AGP >1 g/L** |  |  |  |  |
| Baseline | 29 (10.5) | 33 (11.3) | 0.92 (0.54-1.56) | 0.75 |
| End study | 34 (12.2) | 27 (9.2) | 1.37 (0.81-2.34) | 0.24 |

**Supplementary Table 3: Baseline comparison for SES and other variables among two groups for the subset contributing to study**

|  | **Fortified Yoghurt** | **Non fortified Yoghurt** |
| --- | --- | --- |
|  | **(n=278)** | **(n=293)** |
| Gender |  |  |
| Boys | 42.2 | 43.1 |
| Type of house |  |  |
| Kacha | 69.1 | 74.1 |
| Pucca | 10.8 | 10.9 |
| Kacha-Pucca | 20.1 | 15.0 |
| Own House | 96.4 | 91.5 |
| Family Type |  |  |
| Nuclear | 91.4 | 93.5 |
| Mother age | 31.66±6.4 | 31.40±6.0 |
| (mean±SD) |  |  |
| Mother Education |  |  |
| Illiterate | 24.5 | 25.3 |
| Primary | 39.6 | 38.6 |
| Secondary | 27.0 | 23.5 |
| College | 9.0 | 12.6 |
| Father Education |  |  |
| Illiterate | 26.2 | 27.4 |
| Primary | 33.8 | 35.8 |
| Secondary | 19.1 | 16.7 |
| College | 20.9 | 20.1 |
| Mother employment |  |  |
| Homemaker | 93.9 | 93.2 |
| Father Employment |  |  |
| Business | 36.7 | 39.6 |
| Farming | 25.5 | 22.9 |
| Father Income (in |  |  |
| Rupees) |  |  |
| No Income | 0.4 | 1.0 |
| 1000-2000 | 23.4 | 23.9 |
| 3000-5000 | 55.0 | 54.6 |
| 6000-10000 | 12.6 | 11.9 |
| >10000 | 7.9 | 7.2 |

All values are in percentage unless specified

**Supplementary Table 4: Comparison for blood parameters between fortified and non-fortified yoghurt groups at end-line**

| **Variable** | **Fortified Yoghurt**  **(n=278)**  **Mean±SD** | **Non Fortified Yoghurt**  **(n=293)**  **Mean±SD** | **Mean diff (95% CI)** | **p value** |
| --- | --- | --- | --- | --- |
| WBC | 9.53±2.90 | 9.77±3.01 | -0.24 (-0.72 – 0.25) | 0.34 |
| Hgb | 11.55±0.82 | 11.40±0.93 | 0.15 (0.005 – 0.29) | 0.04 |
| PLT | 334.13±76.12 | 336.01±76.37 | -1.88 (-14.40 – 10.63) | 0.77 |
| RDW | 13.58±0.99 | 13.63±1.39 | -0.06 (-0.26 – 0.14) | 0.57 |
| Ferritin | 67.78±36.03 | 71.52±39.91 | -3.74 (-9.98 – 2.51) | 0.24 |
| Stfr | 5.68±1.09 | 5.77±1.23 | -0.09 (-0.28 - 0.11) | 0.38 |
| Body Iron Store | 7.02±2.08 | 7.06±2.35 | -0.05 (-0.41 – 0.32) | 0.80 |
| RBP | 1.24±0.28 | 1.16±0.25 | 0.08 (0.03 – 0.12) | 0.00 |
| Zinc | 69.03±23.65 | 68.48±20.70 | 0.55 (-3.10 – 4.19) | 0.77 |
| Iodine | 113.64±94.82 | 99.51±93.55 | 14.14 (-2.07 – 30.34) | 0.09 |
| AGP | 0.77±0.19 | 0.77±0.18 | 0.003 (-0.03 – 0.03) | 0.83 |
| CRP (Pos) | 253 (91.0) | 269 (90.9) |  |  |
| CRP | 0.62±1.87 | 0.75±2.55 | -0.13 (-0.52 – 0.25) | 0.50 |

**Supplementary Table 5: Concentrations of biomarkers in those with normal and elevated acute phase proteins at baseline**

| Variable | Fortified yoghurt | | Non-fortified yoghurt | |
| --- | --- | --- | --- | --- |
|  | HHHealthy  mean ± SD | Inflammation  mean ± SD | Healthy  mean ± SD | Inflammation  mean ± SD |
|  | n = 249 | n = 29 | n = 259 | n = 33 |
| Hemoglobin (g/L) | 114.59 ± 8.7 | 115.24 ± 7.5 | 115.17 ± 8.3 | 110.64 ± 11.2 |
| Ferritin (µg/L) | 67.07 ± 34.2 | 78.61 ± 48.2 | 69.02 ± 38.7 | 77.98 ± 43.3 |
| sTfR (mg/L) | 5.67 ± 1.2 | 5.84±1.01 | 5.71 ± 1.2 | 6.47± 1.6 |
| Body iron store (mg/Kg body wt) | 6.99 ± 2.2 | 7.31 ± 2.4 | 7.02 ± 2.2 | 6.80 ± 2.5 |
| RBP (µmol/L) | 1.26 ± 0.3 | 1.20± 0.3 | 1.24 ± 0.3 | 1.10 ± 0.3 |
| Zinc (µmol/L) | 9.13± 3.4 | 9.03 ± 2.3 | 9.35 ± 3.7 | 8.28 ± 2.6 |
| Iodine (µg/L) | 155.06 ± 92.3 | 210.94 ± 86.6 | 182.8± 102.4 | 179.62 ± 108.8 |

**Supplementary Table 6: Concentrations of biomarkers in those with normal and elevated acute phase proteins at end-line**

| Variable | Fortified yoghurt | | Non-fortified yoghurt | |
| --- | --- | --- | --- | --- |
|  | HHHealthy  mean ± SD | Inflammation  mean ± SD | Healthy  mean ± SD | Inflammation  mean ± SD |
|  | n = 244 | n = 34 | n = 268 | n = 27 |
| Hemoglobin (g/L) | 115.52 ± 8.4 | 115.21 ± 7.0 | 114.2 ± 9.2 | 111.6 ± 10.8 |
| Ferritin (µg/L) | 64.81 ± 33.1 | 89.13 ± 47.7 | 70.19 ± 40.4 | 84.83 ± 32.6 |
| sTfR (mg/L) | 5.67 ± 1.1 | 5.77 ± 1.1 | 5.73 ± 1.23 | 6.10 ± 1.17 |
| Body iron store (mg/Kg body wt) | 6.90 ± 1.9 | 7.84 ± 2.8 | 6.99 ± 2.4 | 7.81 ± 1.66 |
| RBP (µmol/L) | 1.26 ± 0.3 | 1.11 ± 0.2 | 1.18 ± 0.24 | 0.99 ±0.28 |
| Zinc (µmol/L) | 10.55 ± 3.6 | 10.62 ± 3.9 | 10.50 ± 3.18 | 10.15 ± 3.04 |
| Iodine (µg/L) | 114.0 ± 95.2 | 110.9 ± 93.5 | 98.2 ± 95.3 | 114.3 ± 75.0 |
